# Role of gut microbiota in the pathogenesis of metabolic syndrome: an updated comprehensive review from mechanisms to clinical implications

**DOI:** 10.1097/MS9.0000000000003656

**Published:** 2025-07-29

**Authors:** Ajeet Singh, Amogh Verma, Saad Ashraf, Danish Sarfraz Sheikh, Hamza Irfan, Rumaisa Riaz, Fnu Venjhraj, Shehdev Meghwar, Ravesh Kumar, Muhammad Daoud Tariq, Hafiz Muhammad Hamza, Areeba Ahsan, Prakasini Satapathy

**Affiliations:** a Department of Internal Medicine, Dow University of Health Sciences, Karachi, Pakistan; b Department of Internal Medicine, Rama Medical College Hospital and Research Centre, Hapur, Uttar Pradesh, India; c Department of Medicine, Dow University of Health Sciences, Karachi, Pakistan; d Department of Internal Medicine, Foundation University Medical College, Islamabad, Pakistan; e Department of Medicine, Shaikh Khalifa Bin Zayed Al Nahyan Medical and Dental College, Lahore, Pakistan; f Department of Internal Medicine, Shaheed Mohtarma Benazir Bhutto Medical College Lyari, Karachi, Pakistan; g Department of Internal Medicine, Liaquat University of Medical & Health Sciences, Jamshoro, Sindh, Pakistan; h Department of Internal Medicine, Foundation University Medical College, Islamabad, Pakistan; i Foundation University School of Health Sciences, Islamabad, Pakistan; j Department of Medicine, Foundation University Medical College, Islamabad, Pakistan; k University Center for Research and Development, Chandigarh University, Mohali, Punjab, India; l Medical Laboratories Techniques Department, AL-Mustaqbal University, Hillah, Babil, Iraq

**Keywords:** diabetes mellitus, gut microbiota, metabolic syndrome, microbiome-heart axis, obesity

## Abstract

Modern studies have linked gut microbiota to metabolic syndrome – a condition linked to obesity, characterized by insulin resistance, dyslipidemia, hyperglycemia, and hyperlipidemia. The gut microbiota, influenced by diet, plays a pivotal role in metabolic syndrome, affecting energy absorption, metabolism, and immune responses. Dysbiosis disrupts energy metabolism and immune responses contributing to metabolic endotoxemia, leading to insulin resistance and systemic inflammation. Key metabolites like short-chain fatty acids and bile acids, modulate insulin sensitivity and metabolic pathways. Therapeutic strategies involving probiotics and prebiotics show potential in managing diabetes and cardiovascular diseases by targeting lipid metabolism, inflammation, and atherosclerosis. However, challenges in therapy standardization and regulatory approval remain. Continued research on gut microbiota’s role in metabolic syndrome could lead to innovative, personalized treatment and prevention strategies based on individual metabolic profiles. The review aims to elucidate the underlying mechanisms that influence metabolic health and cardiovascular function. It seeks to synthesize current research findings, highlighting the role of microbial composition, diversity, and metabolic byproducts in the modulation of host metabolism and cardiovascular outcomes.

## Introduction

Metabolic syndrome is a collection of metabolic abnormalities associated with obesity that increase the risk of cardiovascular disease (CVD) and type 2 diabetes. Key components of this syndrome are insulin resistance, dyslipidemia, hyperglycemia, and hyperlipidemia^[1,2]^. Metabolic syndrome models using humans and animals have indicated the microbiota of the gut as a major component of the illness. Gut microorganisms are highly sensitive to diet compositions, so there is a drastic alteration in their makeup and functionality. In influencing the host’s metabolic status through controlled energy intake and disposition as well as hunger signals and metabolism of carbohydrates and proteins and lipids and storage and intermediary metabolism and detoxification, the intestinal “superorganism,” integrates gut motility and energy absorption^[3,4]^. The bacterial fragments, particularly lipopolysaccharides (LPS), may translocate from the gut into the bloodstream when illness disrupts the homeostasis between bacterial pathogens and the host’s immune system. This state, known as “metabolic endotoxemia,” occurs when elevated levels of endotoxins in the circulation contribute to the development of insulin resistance and systemic inflammation[3]. Increased food intake and/or decreased physical activity are the primary causes of nutrient excess linked to the rising incidence of metabolic syndrome[4]. According to a recent study, there is a strong correlation between gut microbiota and disorders connected to metabolic syndrome. Even in the context of obesity, gut microbiota plays a crucial role in regulating nutrition by influencing energy balance. It specifically affects triglyceride storage and the breakdown of fatty acids, which are essential processes for releasing stored energy. The rather complex interplay can indeed directly alter multiple metabolic processes and regulators within the host gene in mice models. Despite reduced food consumption and a 40% decrease in muscle mass, studies in humans have shown that alterations in gut microbiota can lead to significant increases in body fat and insulin resistance highlighting microbiota’s role in regulating metabolic processes[4]. This review adds to the existing literature by synthesizing recent findings on the mechanistic links between gut microbiota and the pathophysiology of metabolic syndrome, emphasizing pathways such as metabolic endotoxemia, energy regulation, and host gene modulation. Key learning points include the role of gut microbiota in lipid metabolism, insulin sensitivity, and systemic inflammation, and how targeted microbiome interventions could represent novel strategies for mitigating metabolic syndrome. In line with current best practices for transparency in scientific reporting, particularly regarding the use of artificial intelligence tools, this review adheres to the TITAN 2025 Guidelines[5].

## Gut microbiota composition and diversity

While the number of bacterial species in the human stomach can differ substantially depending on the research, it can range between 500 and 1000 only. Still, a current experiment that was conducted with multiple participants suggested that over 35 000 various kinds of bacteria can be found in the human gut microbiota[6]. The components of the gut microbiota include viruses, yeast, and bacteria that comprise several species of microorganisms. Bacteria in the gut are classified into several phyla, with six main groups representing 90% of the gut microbiota: Firmicutes, Bacteroidetes, Actinobacteria, Proteobacteria, Fusobacteria, and Verrucomicrobia. Among these, Firmicutes and Bacteroidetes dominate. The Firmicutes phylum includes genera like Lactobacillus, Bacillus, and Clostridium, with Clostridium making up 95% of Firmicutes. Bacteroidetes are mainly composed of the genera Prevotella and Bacteroides, while Actinobacteria are primarily represented by Bifidobacterium^[6,7]^.

**Figure 1.**
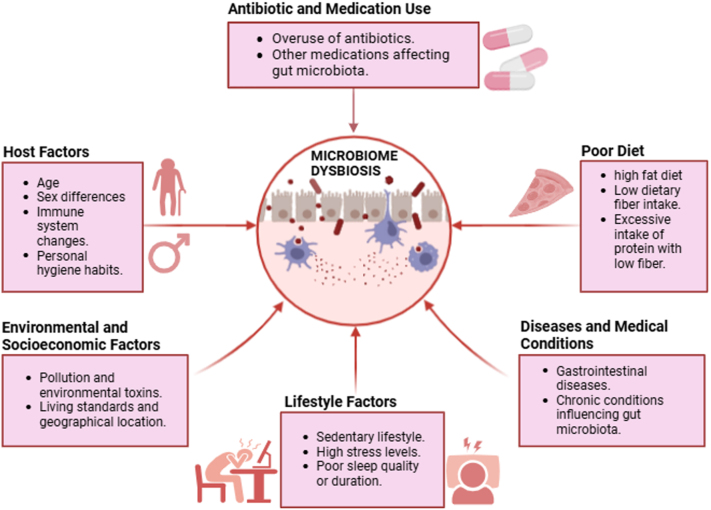
Environmental and lifestyle risks leading to gut dysbiosis.

Several factors determine the composition of the microbial communities in the gut. Diet is important as does the kind and quantity of food being ingested influence the composition of the microorganisms regarding the amounts of carbohydrates, lipids, and fiber to mention but a few[7]. Antibiotics and other drugs that patients may take will reduce strains of beneficial bacteria in the body and alter the composition of gut microbiota[8]. In the aspect of optimizing the population of beneficent bacteria, the role of probiotics and prebiotics is that they are beneficial for the modulation of gut microbiota. It is also found that life has a role to play concerning gut microbiota alterations, and the changes are observed from childhood to elderly life and the difference of sex^[8,9]^. The microbial population is affected by some factors such as living standards, pollution, geographical location, and host immunity. Such variables include stress levels, sleep duration, and quality, patterns of physical activity, and others that have an impact on the individuals’ gut flora. Besides, diseases including gastrointestinal diseases, and personal hygienic habits can greatly affect the levels of the different types of gut bacteria[10]. Figure 1 illustrates the environmental and lifestyle risks leading to gut dysbiosis. Various techniques have advanced the study of gut microbiota and its role in metabolic syndrome. Metagenomics and 16S rRNA sequencing have been instrumental in identifying microbial compositions linked to obesity and insulin resistance. Metatranscriptomics provides insight into gene activity, revealing functional changes in microbial metabolism. Culturomics has expanded our understanding by isolating previously uncultured microorganisms, while metaproteomics analyzes microbial proteins that influence host metabolic processes. These have collectively deepened our knowledge of gut microbiota’s contribution to metabolic disorders, helping to identify potential therapeutic targets. When combined, these methods offer a comprehensive grasp of the composition, roles, and traits of the gut microbiota^[11,12]^. Gut microbiota stability and resilience are essential for sustaining metabolic homeostasis and determining the success of long-term therapeutic interventions. Stability refers to the microbial community’s ability to maintain its structure over time, while resilience describes its capacity to recover from external disturbances such as antibiotics, dietary shifts, or infections. Factors like host genetics, mode of birth delivery, early-life nutrition, long-term dietary patterns, and environmental exposures play a major role in shaping these traits. For instance, diets rich in fermentable fibers and polyphenols have been shown to enhance microbial diversity and promote resilience by fostering beneficial taxa capable of restoring ecological balance following disruption[10]. A more diverse gut microbiota has also been linked to increased resistance to pathogen colonization and greater capacity for functional recovery after perturbations[11]. Understanding these parameters is key for assessing the long-term viability of microbiome-targeted therapies in metabolic syndrome.

**HIGHLIGHTS**

- Gut microbiota plays a critical role in metabolic processes, affecting energy intake, hunger regulation, metabolism, and systemic inflammation. Disruption of gut microbiota leads to metabolic endotoxemia, which contributes to insulin resistance and inflammation.
- Methods for studying microbiota include metagenomics, 16S rRNA sequencing, metatranscriptomics, culturomics, and metaproteomics, offering a comprehensive understanding of the microbial community.
- Regular exercise positively affects gut microbiota composition, improving metabolic profiles and insulin sensitivity. Combined with a specific diet, exercise can enhance microbial diversity and overall host health.

## Gut microbiota and obesity

### Evidence linking gut microbiota to obesity

Gut microbiota play a crucial role in obesity, making them a key research focus for potential treatments[13]. Notably, diet significantly influences gut microbiota composition, affecting obesity risk. Studies in humans and mice suggest that certain bacteria, including Firmicutes, Bacteroidetes, Rhizobium, Lactococcus, and Clostridium, contribute to obesity by increasing energy harvest, producing short-chain fatty acids (SCFAs), and inducing low-grade inflammation^[13–15]^. The gut microbiota, which contains approximately three million genes – far exceeding the human genome – enhances metabolic functions in the gut mucosa^[16,17]^.

**Figure 2.**
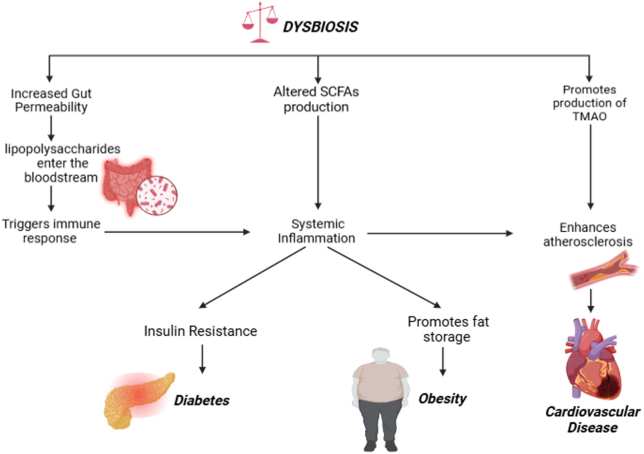
Exploring the pathways from gut microbiota to obesity, diabetes, and cardiovascular risk.

Several pathways link gut microbiota to obesity development. One mechanism involves the inhibition of adenosine monophosphate kinase (AMPk), an enzyme regulating cellular energy. Suppression of AMPk reduces fatty acid oxidation, leading to increased fat storage^[18,19]^. Additionally, gut microbiota contributes to systemic inflammation, further disrupting metabolism[20]. Systemic inflammation refers to a widespread inflammatory response throughout the body, often characterized by elevated levels of pro-inflammatory cytokines (e.g. TNF-α, IL-6) in the circulation. Research has shown that gut microbiota enhances energy extraction from food. Turnbaugh *et al*[21] found that obese individuals harbor microbiota with a higher capacity for breaking down complex carbohydrates, increasing energy absorption due to a higher Firmicutes-to-Bacteroidetes ratio. Moreover, gut bacteria ferment dietary fibers into SCFAs, which contribute up to 10% of daily caloric intake and stimulate appetite-regulating hormones like peptide YY (PYY) and glucagon-like peptide-1 (GLP-1), influencing energy balance[22]. Gut microbiota influence adipose tissue metabolism and inflammation through bioactive metabolites, gut barrier modulation, and gene regulation^[23,24]^. Conversely, gut dysbiosis can lead to increased intestinal permeability, allowing bacterial endotoxins like LPS to enter the bloodstream^[25,26]^. This “metabolic endotoxemia” triggers chronic inflammation and insulin resistance, contributing to obesity[27]. Figure 2 illustrates various mechanisms through which gut dysbiosis contributes to the development of obesity, diabetes mellitus, and CVDs (Table 1).

**Table 1:** Key studies on gut microbiota and obesity

Aspect	Study	Findings	Outcomes
Energy harvesting	Turnbaugh *et al* (2006)	Increased energy extraction in obese microbiota	Higher Firmicutes-to-Bacteroidetes ratio enhances carbohydrate breakdown and calorie absorption.
	Bäckhed *et al* (2004)	Gut microbiota promotes fat storage	Colonization of germ-free mice led to a 60% increase in body fat despite similar food intake.
Short-chain fatty acids (SCFAs)	May *et al* (2021)	Butyrate prevents diet-induced obesity	Butyrate enhanced mitochondrial function, increased energy expenditure, and reduced inflammation in adipose tissue.
	Den Besten *et al* (2013)	SCFAs regulate appetite and metabolism	SCFAs stimulate the release of appetite-regulating hormones (PYY, GLP-1) and provide up to 10% of daily energy.
Inflammation and gut barrier	Cani *et al* (2007)	Metabolic endotoxemia linked to obesity	Elevated lipopolysaccharides (LPS) levels increased inflammation, disrupted gut barrier, and promoted fat storage.
Obesogenic microbiota	Clarke *et al* (2012)	Microbial shifts in obese individuals	Obesity is associated with reduced Bacteroidetes and increased Firmicutes, enhancing dietary energy harvest.
	David *et al* (2014)	Diet-induced gut microbiota changes	High-fat diets increase bile-tolerant bacteria and inflammation; plant-based diets enhance SCFA-producing bacteria.
Dietary interventions	Beam *et al* (2021)	Calorie-restricted diets improve gut microbiota composition	Increased Bacteroidetes, decreased Firmicutes, and improved metabolic health markers.

### Impact of obesity on gut microbiota composition

Obesity not only is influenced by gut microbiota but also affects its composition, creating a bidirectional relationship. A multitude of data points to the gut microbiota’s role in the emergence of obesity and related comorbidities[14]. Obesity is associated with reduced microbial diversity and an altered abundance of specific bacterial taxa. Clarke *et al*[28] reported that obese individuals have a lower proportion of Bacteroidetes and a higher proportion of Firmicutes compared to lean individuals. This shift in the microbial community is thought to increase the capacity for energy harvest from diet[28]. David *et al*[29] conducted a study where participants switched between plant-based and animal-based diets. The animal-based diet, rich in fats and proteins, rapidly altered the gut microbiota composition, increasing the abundance of bile-tolerant bacteria such as Alistipes and Bilophila, while decreasing the levels of SCFA-producing bacteria. These changes were associated with increased microbial metabolic activity and inflammation[29]. Despite the impact of obesity on gut microbiota, dietary interventions can partially restore microbial diversity and function. A study by Beam *et al*[30] showed that a calorie-restricted diet in obese individuals led to significant changes in gut microbiota composition, including increased Bacteroidetes and decreased Firmicutes, along with improved metabolic health markers. Obesity-induced changes in gut microbiota also affect bile acid metabolism. Bile acids are synthesized from cholesterol in the liver and play a key role in fat digestion and absorption. Gut bacteria convert primary bile acids into secondary bile acids, which can influence lipid metabolism and energy homeostasis. Changes in gut microbiota composition in obesity can alter the bile acid pool, affecting host metabolism and contributing to metabolic dysfunction[31].

## Gut microbiota and diabetes mellitus

### Relationship between gut microbiota and insulin resistance

#### Mechanisms of gut microbiota-induced insulin resistance

According to recent research, changes in the gut microbiota’s composition are strongly associated with the development of diabetes^[32,33]^. One of the main factors influencing the gut microbiota’s makeup and a major contributing factor to the onset of diabetes is diet[34]. Insulin resistance, a condition where cells in the body become less responsive to insulin, is a key feature of type 2 diabetes mellitus (T2DM)[35]. The gut microbiota affects intestinal permeability, and an imbalance (dysbiosis) can lead to increased permeability. This condition allows LPS from Gram-negative bacteria to enter the bloodstream, causing metabolic endotoxemia. LPS activates TLR4, inducing a chronic inflammatory state that interferes with insulin signaling pathways[36]. Cani *et al* demonstrated that metabolic endotoxemia is linked to low-grade inflammation and insulin resistance[28]. Increased intestinal permeability is observed in individuals with T2DM, contributing to systemic inflammation and insulin resistance[37]. Certain gut microbial metabolites, like trimethylamine-N-oxide (TMAO), are implicated in insulin resistance. TMAO is produced from dietary choline and carnitine by gut bacteria and further metabolized in the liver. Elevated TMAO levels are associated with increased insulin resistance and a higher risk of CVDs, DM, and cancers[38]. Research suggests that changes in butyrate and incretin secretions may be a mediating factor in the relationship between microbiome and type 2 diabetes[39] (Table 2).

**Table 2:** Key studies on gut microbiota and diabetes

Aspect	Study	Findings	Outcomes
Gut microbiota and insulin resistance	Cani *et al* (2007)	Metabolic endotoxemia linked to insulin resistance	Elevated LPS levels increase inflammation, disrupting insulin signaling and contributing to insulin resistance.
	Gurung *et al* (2020)	Dysbiosis and increased gut permeability in T2DM	Dysbiosis increases intestinal permeability, allowing endotoxins to trigger chronic inflammation.
Role of SCFAs	Portincasa *et al* (2022)	SCFAs regulate glucose and lipid metabolism	SCFAs enhance insulin sensitivity by activating GPCRs (e.g. GPR41, GPR43) and modulating glucose homeostasis.
	Tang *et al* (2021)	Butyrate’s role in glucose regulation	Butyrate activates anti-inflammatory pathways and improves insulin sensitivity.
Bile acid metabolism	Ding *et al* (2015)	Bile acids influence glucose metabolism	Bile acids activate FXR and TGR5 receptors, improving glucose homeostasis and insulin sensitivity.
	Thomas *et al* (2009)	GLP-1 release mediated by TGR5 activation	Bile acid signaling enhances GLP-1 secretion, improving glucose tolerance and insulin secretion.
Therapeutic approaches	Vrieze *et al* (2012)	FMT improves insulin sensitivity	Fecal microbiota transplantation from lean donors increased butyrate-producing bacteria and improved insulin action.
	Paone *et al* (2022)	Prebiotics modulate gut microbiota to improve metabolic outcomes	Oligofructose increased SCFA production, reduced endotoxemia, and improved glucose tolerance.
	Liu *et al* (2022)	Akkermansia muciniphila as a therapeutic probiotic	Enhances gut barrier function, reduces fat mass, and improves insulin sensitivity.
Gut-derived metabolites	Li *et al* (2022)	TMAO linked to insulin resistance and diabetes	Elevated TMAO levels impair insulin signaling and increase diabetes risk.

#### Role of SCFAs and bile acids

SCFAs and bile acids are crucial metabolites produced by gut microbiota that significantly affect insulin sensitivity. They modulate several metabolic pathways and are involved in obesity, insulin resistance, and type 2 diabetes[40]. SCFAs, including acetate, propionate, and butyrate, are produced by the fermentation of dietary fibers. They serve as an energy source for colonocytes and influence systemic metabolism. SCFAs enhance insulin sensitivity by activating G-protein-coupled receptors (GPCRs) such as GPR41 and GPR43, which regulate glucose metabolism and fat storage[41]. Bile acids are synthesized from cholesterol in the liver and aid in fat digestion. Gut bacteria convert primary bile acids into secondary bile acids, which influence metabolic signaling pathways. Bile acids act through the farnesoid X receptor (FXR) to regulate bile acid synthesis, glucose metabolism, and lipid homeostasis. FXR activation improves insulin sensitivity by regulating genes involved in gluconeogenesis and lipid metabolism[42]. Bile acids also activate the TGR5 receptor, which stimulates the release of GLP-1 from enteroendocrine cells. GLP-1 enhances insulin secretion and improves glucose homeostasis[43].

### Gut microbiota modulation as a therapeutic approach for diabetes

Given the significant role of gut microbiota in insulin resistance and T2DM, modulating the gut microbiota presents a promising therapeutic strategy[44]. Gut microbiota influence the endocannabinoid system, which regulates energy balance and glucose metabolism. Modulation of gut microbiota with specific probiotics, such as Akkermansia muciniphila, has been shown to improve gut barrier function, reduce fat mass, and enhance insulin sensitivity[45]. Prebiotics are non-digestible fibers that promote the growth of beneficial gut bacteria. Prebiotic supplementation can improve metabolic health by increasing the production of SCFAs and modulating gut microbiota composition. Oligofructose supplementation improved glucose tolerance and reduced body weight gain in obese mice by increasing the abundance of Bifidobacteria and reducing endotoxemia[46]. Fecal Microbiota Transplantation (FMT) involves transferring fecal material from a healthy donor to the recipient’s gastrointestinal tract. Vrieze *et al*[47] showed that FMT from lean donors to individuals with metabolic syndrome improved insulin sensitivity and increased the abundance of butyrate-producing bacteria. Some studies suggest that electively targeting specific bacterial populations with antibiotics can alter gut microbiota composition[48]. However, a randomized controlled trial (RCT) concluded that the bacterial diversity and Firmicutes involved in the metabolism of bile acid and SCFAs were reduced by vancomycin, but not by amoxicillin. This was accompanied by changes in the amounts of plasma and/or fecal metabolites. Antibiotics increased the expression of oxidative pathways in adipose tissue, while vancomycin decreased the expression of immune-related pathways. Clinical trials investigating gut microbiota modulation have provided promising evidence for its therapeutic potential in T2DM. A randomized controlled trial by Vrieze *et al*[47] demonstrated that FMT from lean donors significantly improved insulin sensitivity in individuals with metabolic syndrome, attributed to increased abundance of butyrate-producing bacteria. Similarly, supplementation with *Akkermansia muciniphila*, a mucin-degrading bacterium, improved glucose homeostasis, reduced body weight, and strengthened intestinal barrier function in overweight or obese subjects[45]. Another study reported that prebiotic oligofructose supplementation enhanced glucose tolerance and reduced endotoxemia in obese mice by modulating gut microbial composition and promoting bifidobacterial growth[46]. These findings underscore the translational relevance of gut microbiota manipulation as an adjunct strategy for glycemic control. Tissue-specific insulin sensitivity, energy/substrate metabolism, postprandial hormones and metabolites, gut permeability, systemic inflammation, and adipocyte size were unaffected by antibiotics[49].

## Gut microbiota and CVD

### Gut microbiota and lipid metabolism

Gut microbiota play a crucial role in cholesterol metabolism and cardiovascular health. Certain bacteria, such as *Lactobacillus* and *Bifidobacterium*, aid cholesterol absorption by deconjugating bile salts, while others, like *Eubacterium coprostanoligenes*, convert cholesterol into coprostanol, a poorly absorbed form that lowers serum cholesterol levels[50]. Clinical studies support the role of probiotics in reducing total cholesterol and LDL levels, thereby decreasing the risk of CVDs[51].

Hypercholesterolemia, a key driver of atherosclerosis, is prevalent in metabolic syndrome and is exacerbated by gut microbiota dysbiosis[52]. Alterations in microbial composition influence lipid metabolism by modulating bile acid synthesis and cholesterol absorption. FMT and microbiome profiling studies have confirmed that gut dysbiosis can lead to lipid imbalances that heighten CVD risk^[53,54]^. Additionally, bile acids regulate lipid and glucose metabolism through FXR and TGR5 signaling. Disruptions in these pathways contribute to metabolic dysfunction and atherosclerosis, making them potential therapeutic targets^[55–57]^.

Beyond cholesterol metabolism, microbial metabolites such as trimethylamine N-oxide (TMAO) link gut dysbiosis to CVD^[58,59]^. Elevated TMAO levels correlate with increased risk of major adverse cardiovascular events, including myocardial infarction and stroke, by promoting arterial cholesterol deposition and impairing reverse cholesterol transport^[59–61]^. Additionally, microbial-derived propionate has been shown to reduce blood pressure and protect against hypertensive damage by modulating the sympathetic nervous system and immune function[62]. Secondary bile acids further influence lipid metabolism through TGR5 activation, enhancing energy expenditure and reducing atherosclerosis progression[63]. Other than its established role in lipid metabolism and systemic inflammation, gut microbiota also exerts a regulatory influence on vascular tone and blood pressure. Microbial metabolites such as SCFAs, particularly propionate and butyrate, have been shown to lower blood pressure by activating GPCRs (e.g. GPR41) and modulating sympathetic nervous system activity[62]. Propionate has also been reported to reduce hypertensive damage through immune modulation and improved endothelial function[64]. Additionally, dysbiosis-induced increases in TMAO levels have been linked to impaired vascular reactivity and endothelial dysfunction, both key contributors to atherosclerosis and hypertension[60]. Thus, maintaining a balanced gut microbiota may offer cardioprotective effects via multiple mechanisms, including modulation of vascular homeostasis.

### Role of gut microbiota in inflammation and atherosclerosis

Metabolic endotoxemia triggers systemic inflammation by elevating LPS levels, which activate immune cells and promote the release of pro-inflammatory cytokines. This contributes to insulin resistance and endothelial dysfunction, both of which are linked to atherosclerosis^[65,66]^. SCFAs produced by gut bacteria can reduce inflammation by promoting the development of regulatory T cells^[67,68]^. Gut microbiota composition also influences atherosclerosis risk through mechanisms such as lipid metabolism and immune modulation. For instance, individuals with atherosclerosis exhibit distinct gut microbiota profiles, with increased levels of inflammatory bacteria and alterations in lipid metabolism^[69,70]^. Additionally, patients with CVDs often display gut dysbiosis, with a higher abundance of pathogenic bacteria and a reduced number of beneficial butyrate-producing bacteria^[70–73]^. Modulating the gut microbiota through interventions like FMT and probiotics has shown promise in reducing inflammation and improving lipid metabolism, potentially offering therapeutic benefits for cardiovascular health^[74,75]^ (Table 3).

**Table 3:** Key studies on gut microbiota and cardiovascular disease

Aspect	Study	Findings	Outcomes
TMAO and atherosclerosis	Wang *et al* (2011)	Role of TMAO in atherosclerosis	Elevated TMAO levels promote cholesterol deposition in arterial walls and reduce reverse cholesterol transport.
	Heianza *et al* (2017)	TMAO as a predictor of adverse cardiovascular events	High plasma TMAO levels are associated with increased risk of myocardial infarction, stroke, and mortality.
SCFAs and blood pressure	Bartolomaeus *et al* (2019)	Propionate’s role in hypertension	Propionate supplementation reduced blood pressure and protected against hypertensive organ damage via immune modulation.
Bile acid metabolism	Porez *et al* (2012)	Bile acids regulate lipid metabolism and protect against atherosclerosis.	FXR and TGR5 activation by bile acids improve lipid homeostasis and glucose metabolism, reducing atherosclerosis risk.
	Staley *et al* (2017)	Gut bacteria modulate bile acid pool composition	Altered bile acid metabolism influences cholesterol homeostasis and cardiovascular health.
Inflammation and endotoxemia	Cani *et al* (2007)	LPS-induced systemic inflammation and atherosclerosis.	Elevated LPS levels activate TLR4, triggering pro-inflammatory cytokines and promoting endothelial dysfunction.
	Furusawa *et al* (2013)	SCFAs reduce systemic inflammation.	Butyrate enhances regulatory T cell differentiation, reducing inflammation and atherosclerotic risk.
Gut microbiota dysbiosis	Karlsson *et al* (2012)	Dysbiosis in individuals with atherosclerosis.	Increased Enterobacteriaceae and reduced Bacteroidetes linked to systemic inflammation and lipid metabolism dysregulation
	Jie *et al* (2017)	Microbial enzymes and plaque vulnerability.	Specific microbial enzymes increase TMA production, contributing to plaque formation and coronary artery disease.
Therapeutic approaches	Shimizu *et al* (2015)	Probiotics and cholesterol lowering	Probiotic supplementation reduced LDL cholesterol levels, mitigating cardiovascular risk.
	Nesci *et al* (2023)	FMT as a strategy to reduce atherosclerosis	Fecal microbiota transplantation altered gut microbiota composition and improved lipid metabolism.

## Interaction between gut microbiota, diet, and lifestyle

The composition of gut microbiota is strongly influenced by dietary habits, with high-fat diets and fiber intake playing significant roles. Long-term consumption of a high-fat diet has been associated with reduced microbial diversity and alterations in gut composition, leading to metabolic disturbances. Studies have shown that low-fat diets increase beneficial bacterial populations like *Desulfovibrionaceae* and *Rikenellaceae* RC9, while high-fat diets reduce Lactobacillus and Bifidobacterium counts, which are crucial for maintaining gut health^[76,77]^. Conversely, dietary fiber enhances microbiota richness and supports beneficial bacterial growth, which positively impacts metabolic functions^[78,79]^. The interactions between dietary fatty acids and gut microbiota have been linked to improved metabolic profiles, with certain long-chain saturated fatty acids reducing hepatic steatosis by altering microbial composition^[80–82]^. These findings underscore the importance of diet in shaping gut microbiota and metabolic health.

Beyond diet, lifestyle factors such as physical activity, stress, and sleep also influence gut microbiota. Regular exercise, both short-term and long-term, has been shown to promote beneficial microbiota shifts that improve metabolic health, insulin sensitivity, and glucose homeostasis^[83–85]^. Exercise, particularly when combined with a balanced diet, fosters a gut microbial environment that supports cardiovascular health and may even modulate the risk of metabolic disorders^[86,87]^. Moreover, emerging research suggests that exercise-induced changes in gut microbiota could enhance antitumorigenic properties, potentially contributing to colorectal cancer prevention[88]. However, the impact of exercise varies depending on factors such as intensity, duration, and individual microbiome composition^[89,90]^.

Stress and sleep also play critical roles in gut microbiota regulation. Chronic stress, marked by elevated cortisol levels, has been linked to gut dysbiosis and adverse mental health effects, including anxiety and depression^[91,92]^. Sleep disturbances have been shown to disrupt gut microbiota homeostasis, with studies demonstrating that sleep deprivation can negatively affect intestinal stem cells and microbial regulation via the GABA signaling pathway[93]. Additionally, microbiota-targeted interventions, such as washed microbiota transplantation, have been found to improve sleep quality, highlighting a reciprocal relationship between gut microbiota and sleep[94]. Other interventions, including Ganoderma lucidum administration, have been shown to enhance sleep through microbiota-dependent pathways involving serotonin[95].

## Therapeutic approaches targeting gut microbiota

The modulation of gut microbiota using probiotics and prebiotics has become an increasingly popular therapeutic strategy for improving gut health and managing various diseases, including metabolic syndrome. Probiotics, which are beneficial live microorganisms, and prebiotics, the indigestible dietary fibers that support these microbes, help restore gut microbiota balance and positively influence metabolic functions. Synbiotics, a combination of probiotics and prebiotics, have been shown to be more effective in modulating the microbiota and producing beneficial biological effects than when used individually^[96,97]^. Research indicates that these interventions can influence various physiological processes, such as food intake, body weight, and metabolic functions, providing a potential therapeutic approach for metabolic disorders^[98,99]^. For instance, a study by Sanchez *et al*[100] demonstrated that supplementation with a combination of probiotic strains led to increased GLP-1 levels and reduced BMI in children with NAFLD, supporting the role of probiotics in metabolic regulation. In addition, probiotics and prebiotics have been shown to impact mental health through the gut-brain axis. Imbalances in gut microbiota, or dysbiosis, have been linked to mental health conditions such as depression and anxiety, with specific bacterial strains like *Firmicutes* and *Bacteroidetes* playing key roles^[101,102]^. Probiotics, often referred to as “psychobiotics,” have demonstrated potential in improving mood and neurotransmitter function, which highlights their relevance in managing mental well-being[103]. Furthermore, probiotics have shown promise in alleviating several aspects of metabolic syndrome, including blood pressure, glucose metabolism, lipid profiles, and inflammatory biomarkers[104]. FMT from healthy donors has also shown promising results in improving insulin resistance in individuals with metabolic syndrome, while specific probiotic strains, such as *Lactobacillus rhamnosus GG*, are known to support immune function and maintain intestinal microbial balance, both of which are essential for managing metabolic disorders[105]. While microbiota-based therapies such as probiotics, prebiotics, synbiotics, and FMT show therapeutic promise, several challenges hinder their clinical standardization and regulatory approval. Inter-individual variability in gut microbiota composition, influenced by genetics, diet, age, and geography, complicates the development of universally effective interventions[6]. Moreover, safety concerns remain due to the potential for adverse immune responses, disruption of commensal populations, and transfer of antimicrobial resistance genes[8]. The absence of standardized protocols for microbiome analysis and the lack of long-term safety data further impede regulatory progress[34]. Tailoring microbiome-targeted therapies to individual metabolic and microbial profiles may offer a path forward, but such precision approaches require robust bioinformatics infrastructure and a deeper understanding of host-microbiota interactions.

## Challenges and future directions

Nevertheless, several limitations are inherent to the available literature on the gut microbiota’s involvement in metabolic syndrome. The complexity of gut microbiota-host interactions, involving factors such as energy harvest, butyrate and bile acid modulation, and impacts on various physiological systems, presents significant challenges. The interplay between gut microbiota and metabolic syndrome mechanisms, including toll-like receptors, and the endocannabinoid system, complicates the isolation of specific causal relationships. The heterogeneity of metabolic syndrome, which includes conditions such as insulin resistance, dyslipidemia, hepatic steatosis, and hypertension, poses additional challenges. Understanding the differential contributions of gut microbiota to these conditions is complex. Variability in gut microbiota composition among individuals, influenced by dietary habits, genetics, and environment, complicates the generalization of findings.

Although research has pointed to correlations between the different elements of the gut microbiota and traits of metabolic syndrome, causal relation has yet to be proven by better methods including interventional methods like Mendelian randomization. Additionally, the lack of standardized methodologies for studying the gut microbiota and variations in techniques for microbiome analysis can introduce inconsistencies and hinder the reproducibility of results. The process of translating evidence into practice or clinical uses is a difficult task. Thus, there is some evidence for the use of gut microbiota-targeted therapies in metabolic syndrome. However, the problem lies in the difficulty in developing safe and effective interventions due to several challenges. That include standardization of therapy, prolonged safety concerns, and, most importantly, regulatory approval, remain significant hurdles in developing microbiota-targeted interventions for metabolic syndrome. Standardization of therapy is complicated due to the diversity of gut microbiota among individuals, influenced by factors such as diet, genetics, and environmental exposures, making it difficult to create universally effective treatments. Furthermore, safety concerns arise from the potential for unintended consequences when modifying the gut microbiota, such as the disruption of beneficial microbial communities or the development of antibiotic resistance. Long-term safety data are limited, especially in human clinical trials, making it hard to assess the sustained impact of such interventions. Regulatory approval is also a major challenge, as the current regulatory framework is not well-equipped to evaluate microbiota-based therapies, which are complex and personalized. Regulatory agencies require robust clinical evidence to ensure the efficacy and safety of microbiome-modulating treatments, but the variability in microbiota composition across populations and the lack of standardized methodologies for studying microbiome complicate the generation of such evidence.

Thus, it is possible to state that further investigations of the links between gut microbiota and metabolic syndrome may be beneficial for enhancing the current knowledge and improving the existing approaches. The comprehension of the interactions between gut microbiota and metabolic syndrome and the influence of gut microbiota on other syndromes in metabolic diseases may give directions for further individualized therapy.

## Conclusion

The gut microbiota has emerged as a pivotal player in the pathogenesis of metabolic syndrome and its associated conditions, including insulin resistance, dyslipidemia, and chronic low-grade inflammation. Evidence underscores the influence of gut-derived metabolites on key metabolic pathways, such as glucose homeostasis and lipid metabolism, highlighting the microbiota as a critical determinant of metabolic health. Disruptions in microbial balance not only exacerbate metabolic syndrome but also contribute to cardiovascular complications, emphasizing the need for targeted therapeutic approaches.

Therapeutic modulation of the gut microbiota through probiotics, prebiotics, and dietary interventions offers a promising avenue for addressing metabolic and CVDs. However, challenges remain in translating these findings into clinical practice due to variability in individual microbial profiles, population-specific responses, and limited long-term data. To bridge this gap, future research must adopt a systems biology approach, integrating genomics, metabolomics, and microbiome profiling to unravel the complex interplay between the gut microbiota and host metabolism.

The development of personalized, microbiota-driven therapies tailored to individual metabolic profiles represents a transformative opportunity in the prevention and management of metabolic diseases. By advancing our understanding of gut microbiota-host interactions and leveraging these insights, we can unlock innovative therapeutic strategies that have the potential to reduce the global burden of metabolic disorders and improve population health outcomes. Such progress will mark a paradigm shift in our approach to tackling these complex and multifactorial diseases.

## Data Availability

Not applicable.

## Research registration unique identifying number (UIN)

Research registration is not applicable to this article, as this study does not involve the collection of any data from patients or clinical subjects. It is a narrative synthesis based on existing literature and publicly available data.
